# Siderophore cooperation of the bacterium *Pseudomonas fluorescens* in soil

**DOI:** 10.1098/rsbl.2014.0934

**Published:** 2015-02

**Authors:** Adela M. Luján, Pedro Gómez, Angus Buckling

**Affiliations:** ESI, Biosciences, University of Exeter, Penryn, Cornwall TR10 9FE, UK

**Keywords:** *Pseudomonas fluorescens*, cooperation, soil, natural environment, public goods

## Abstract

While social interactions play an important role for the evolution of bacterial siderophore production *in vitro*, the extent to which siderophore production is a social trait in natural populations is less clear. Here, we demonstrate that siderophores act as public goods in a natural physical environment of *Pseudomonas fluorescens*: soil-based compost. We show that monocultures of siderophore producers grow better than non-producers in soil, but non-producers can exploit others' siderophores, as shown by non-producers' ability to invade populations of producers when rare. Despite this rare advantage, non-producers were unable to outcompete producers, suggesting that producers and non-producers may stably coexist in soil. Such coexistence is predicted to arise from the spatial structure associated with soil, and this is supported by increased fitness of non-producers when grown in a shaken soil–water mix. Our results suggest that both producers and non-producers should be observed in soil, as has been observed in marine environments and in clinical populations.

## 1. Introduction

Despite being one of the most abundant elements in the Earth's crust, iron is a major limiting factor for bacterial growth, because most of the iron in natural habitats is in the insoluble Fe(III) form [1]. In response to iron limitation, microbes have evolved numerous mechanisms to scavenge iron from their surroundings. One mechanism is the facultative production and uptake of extracellular siderophores, low molecular weight ferric-specific ligands [1]. The role of these compounds is to deliver iron into the cell via specific receptor and transport systems. A crucial feature of siderophore production is that it is a form of public goods cooperation under iron limitation: siderophores benefit all bacteria within the locality that are capable of taking up the siderophore–iron complex, but their production is metabolically costly to the producer [2]. This makes siderophore secretion open to invasion by non-producing ‘cheats’, who pay none of the costs of siderophore production [2,3], but can still use siderophores produced by nearby cells.

The involvement of siderophores as a public good cooperative trait in *Pseudomonas* spp. has been well-established both *in vitro* (*P. aeruginosa* and *P. fluorescens*; although only when iron is sufficiently limited [4,5]) and in animal models (references in [6]). However, the relevance of social interactions for the evolution of siderophore production in natural populations is less clear. Studies of clinical populations of *P. aeruginosa* [6] and marine populations of *Vibrio* spp. [7] have identified both siderophore producers and non-producers and hence are consistent with the view (but do not prove) that siderophore production is a public good. In addition, it has been reported that *P. fluorescens*’ pyoverdine synthesis genes are expressed in soil and on plant surfaces [8,9] and that *P. putida* can take up siderophores produced by other rhizosphere microorganisms to regulate its iron status [10].

Here, we investigate whether *P. fluorescens* siderophore production is a public good in a natural physical environment of this bacterium: soil-based compost. We determine whether siderophore production confers a population growth rate benefit, whether non-producers can exploit the siderophore of producers and whether frequency-dependent selection, which is predicted to arise in spatially structured environments [11], operates between producers and non-producers in both structured and unstructured (soil–water mix) environments.

## 2. Material and methods

### (a) Bacterial strains

*Pseudomonas* fluorescens SBW25 [12] was used as the siderophore producer, and strain PBR840, a *pvdL* knockout mutant of SBW25 [13] that does not produce siderophores, was used as the non-producer. It had been previously determined that PBR840 growth was significantly reduced compared with that of the wild-type only under iron-depleted conditions; there was no significant difference in growth between the wild-type and PBR840 strains in iron-rich environments [13].

### (b) Culturing conditions

Microcosms were set up in 9 cm Petri dishes containing 30 g of twice-autoclaved compost soil (Verve Multi-Purpose Compost, UK). Strains were grown overnight in King's medium B (KB; 180 r.p.m., 28°C). Two different soil types differing in their pH range were used in order to manipulate iron availability to the bacteria: acidic soil (70% peat, pH = 4, model: 9751G), where the largest proportion of Fe is in its ferrous form and is expected to be available to the bacteria; and neutral soil (0% peat, pH = 7, model: 9757G), where iron availability is limited, because the Fe(III) form predominates [14]. Soil pH was determined with soil–water suspensions (1 : 1 wt/vol.) using a Jenway 3510 pH meter.

Microcosms were inoculated with approximately 3 × 10^6^ colony forming units (CFU) g^−1^ of either producers, non-producers or a 1 : 1 mixture of both and placed in an environmental chamber at 26°C and 80% humidity. Every 6 days, soil samples (1 g) were collected as previously described [15]. The resultant soil washes were diluted, plated onto KB agar and incubated for 2 days at 28°C to determine CFU g^−1^ of soil [15]. In the mixed cultures, producers and non-producers were differentiated by their green and white colonies, respectively. Six replicates were assayed per treatment, although one of the mixed cultures in neutral soil was discarded. For each time interval, the growth rate of each strain was calculated as *r* = ln(*N*_*f*_/*N*_*i*_), where *N*_*i*_ is the starting density at time *i* and *N*_*f*_ is the final density at time *f*.

### (c) Short-term competition experiments

The relative fitness of non-producers was assessed in neutral soil with the following proportions of non-producers in the initial population: 0.01, 0.5 and 0.99. A total of approximately 3 × 10^6^ CFU g^−1^ of cells was inoculated into 30 ml glass universals containing 3 g of twice-autoclaved soil. The use of tubes allowed a comparison with structured and mixed soil environments (see below). Six replicates were performed for each treatment, and bacterial population densities were determined after 6 days as mentioned above. We calculated the parameter *v* = (*x*_1_(1 − *x*_0_))/(*x*_0_(1 − *x*_1_)) to evaluate the non-producers' fitness, where *x*_0_ and *x*_1_ are the initial and final non-producers' frequencies, respectively. Values of *v* = 1, >1 and <1 indicate equal, higher and lower non-producers’ fitness, respectively [11]. Soil–water mix experiments were carried out by adding 9 ml of sterile double-distilled H_2_O to the respective tubes, and the incubation was performed with constant agitation (180 r.p.m.).

### (d) Statistical analyses

Linear-mixed models, two-way ANOVA and one-sample *t*-tests were employed to assess statistical differences (electronic supplementary material).

## 3. Results

**Figure 1.**
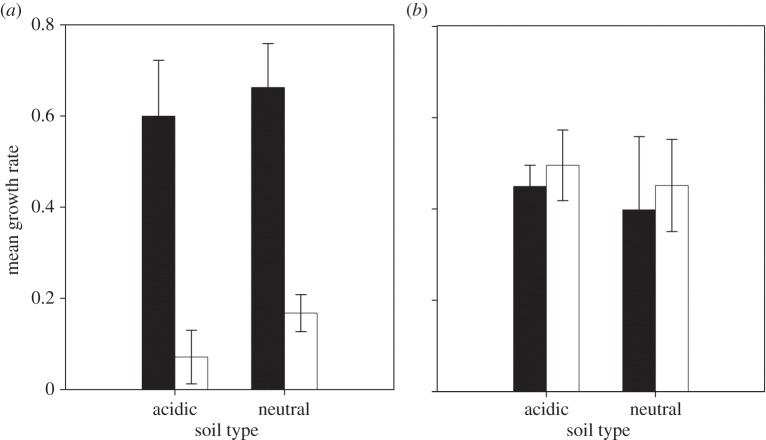
Growth of *P. fluorescens* producers and non-producers in soil microcosms. Bars represent mean values (± s.e.m.) of producer (black) and non-producer (white) growth rates obtained every 6 days over the course of the experiment. Growth rates were determined in monoculture (*a*) or coculture (*b*) acidic and neutral soil microcosms.

We first examined the benefits of siderophore production in *P. fluorescens* bacterial populations in acidic and neutral soils by measuring growth rates of producers and non-producers during 30 days as monocultures. We found that siderophore producers had a higher mean growth rate than non-producers under both soil conditions (figure 1*a*; generalized linear model: *F*_1,21_ = 45.23, *p* < 0.0001), whereas the growth rate of producers and non-producers did not differ between soils (figure 1*a*; *F*_1,21_ = 1.1, *p* > 0.2). This suggests that siderophore production is beneficial for *P. fluorescens* growth in soil, as reported *in vitro* [3]. Note that producers' final cell densities were 3.2 and 2.4 times higher after 30 days of growth than those of non-producers in acid and neutral soils, respectively (electronic supplementary material, S1).

We next investigated whether siderophore production is a costly public good in soil by determining how producers and non-producers influence each other's growth rates. Non-producers showed a significant increase (GLM: *F*_1,20_ = 31.23, *p* < 0.0001) in their growth rate in the presence of producers relative to their growth rate in monoculture (figure 1*a,b*), with no effect of soil type (GLM: *F*_1,20_ = 0.21, *p* > 0.2); the producers' growth rate declined in the presence, versus the absence, of non-producers (GLM: *F*_1,20_ = 4.42, *p* < 0.05), with no effect of soil type (GLM: *F*_1,20_ = 0.007, *p* > 0.2). These results show that siderophore production is an individually costly public good in soil. However, in contrast to the results of *in vitro* studies [3,6,11], we found that non-producers were not able to outcompete producers in soil, where the relative fitness of the non-producers (*v*) was not significantly different from 1 (one-sample *t*-test: acidic *t*_5_ = 1.36, *p* = 0.23; neutral *t*_4_ = 1.03, *p* = 0.36; electronic supplementary material, S2).

**Figure 2.**
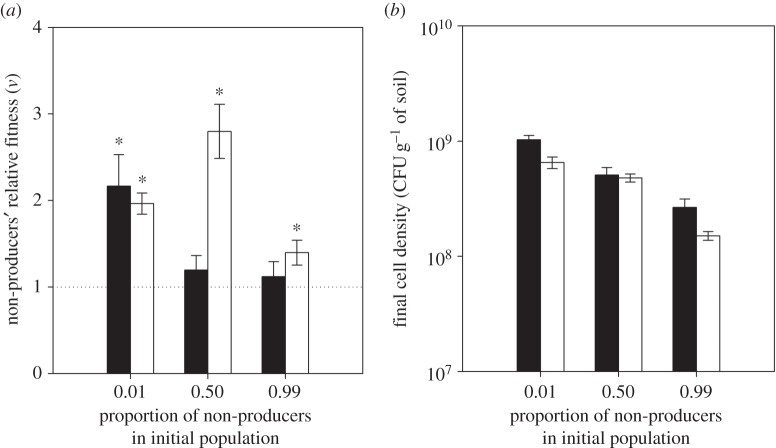
Relative non-producer fitness (*a*) and final population cell density (*b*) at different initial ratios of producers : non-producers in soils differing in spatial structure. Non-producers' relative fitness (*v*; mean ± s.e.m.) and final density (mean ± s.e.m.) were determined after 6 days of growth in static (black) and shaken (white) soil microcosms. Asterisks indicate significant differences (*p* < 0.05) from 1 after Benjamini–Hochberg corrections from multiple comparisons.

To examine whether non-producer fitness was affected by the spatial structure generated by soil [11], we performed experiments competing producers and non-producers at different initial ratios in structured (static) or unstructured (soil–water mix) soil microcosms. After 6 days, we found that non-producers’ relative fitness was higher in the soil–water mix than in soil (figure 2*a*; two-way ANOVA: *F*_1,29_ = 8.78, *p* = 0.006), and the frequency-dependent fitness was affected by whether soil was static or mixed (figure 2*a*; frequency × environment, *F*_2,29_ = 4.5, *p* = 0.019). Specifically, non-producers had a fitness advantage over producers only when initially rare (frequency of 0.01) in soil (figure 2*a*; one-sample *t*-test: *p* = 0.04, corrected for multiple comparisons [16]) but equal fitness to producers when initiated at 0.5 or 0.99. By contrast, non-producers could invade in soil–water mix when initiated at frequencies of both 0.01 and 0.5 (figure 2*a*; 0.01: *p* = 0.003; 0.5: *p* = 0.006, corrected for multiple comparisons [14]). We also found that total population growth was greater in static soil (figure 2*b*; two-way ANOVA: *F*_1,29_ = 9.47, *p* = 0.004) and when non-producers were rare (figure 2*b*; *F*_2,29_ = 61.94, *p* < 0.0001).

## 4. Discussion

Our findings demonstrate that siderophore production by *P. fluorescens* is a cooperative public good in soil compost. As previously established *in vitro* [3], we found a growth rate cost to non-producer relative to producer when populations were grown as monocultures. In addition, non-producer growth rate was increased and producer growth rate decreased when the two were cocultured. However, while non-producers could invade when rare, they could not outcompete producers. Consistent with previous observations [7,17], these results suggest that social interactions affect the evolution of siderophore production in natural populations.

Iron is more soluble and biologically available at low pH [1], and given that siderophore production is regulated in response to iron availability [1], we hypothesized less difference in growth between siderophore producers and non-producers in acidic versus neutral soils. However, we found no effect of soil type. The most likely explanation is that although the solubility of inorganic Fe might be relatively high in acidic soils (approx. 10^−8^ M at pH 4; [16]), it is still far below that required for bacterial growth. Consistent with this view, the presence of high concentrations of siderophores has been reported for a variety of soil–water extracts obtained from soils with different pH ranges [18]. Moreover, it has been established that under aerobic conditions the Fe(III) requirements of the least-demanding microorganism species would be met only at very acidic pH [13]. However, we cannot exclude that the lack of effect of soil type is due to differences other than the availability of iron between the two soil types.

Although we cannot discard potential confounding effects of the addition of water to the soil microcosms, the spatial structure of soil is likely to have contributed to the inability of non-producers to outcompete producers, as shown by an increase in non-producer fitness in the soil–water mix. This is presumably both because spatial structure is likely to limit the diffusibility of siderophores, meaning that producers will preferentially benefit from their own siderophores, and because neighbouring cells that will benefit from siderophores from the focal cell are more likely to be clone mates of the producing cells (i.e. relatedness is high [19,20]). Spatial structure is also predicted to result in negative frequency-dependent fitness and coexistence of producers and non-producers if selection is sufficiently strong [11]. While we observed a fitness advantage for rare non-producers in soil, we were unable to detect an advantage for rare producers. A possible explanation for this asymmetry is that the fitness advantage of non-producers when rare is also driven to some extent by a greater population size when producers are common [11,21], as was observed here at low and intermediate non-producer frequencies. This could also explain why non-producers had higher fitness at both low and intermediate than at high frequencies in the unstructured soil–water mix.

Our results suggest that the evolution of siderophore production can be affected by social interactions in the soil environment, although an important caveat is that we do not consider the impact of the other resident soil microbiota. The ability of non-producers to invade but not outcompete producers suggests both producers and non-producers should be observed in soil, as they have been observed in marine environments [7] and in clinical [6,17] populations. These data add to the small but growing body of literature [7,17,22–24] suggesting that microbial exoproducts are important for social interactions in natural populations (but see Bozdag & Greig [25]).

## Supplementary Material

Supplementary Information

## Supplementary Material

Figure S1: Growth curves of P. flourescens producers and non-producers in mono and mixed soil microcosms.

## Supplementary Material

Figure S2: Relative fitness of non-producers in acidic and neutral soil microcosms.

## Data accessibility

The datasets supporting this article have been deposited in Dryad (http://datadryad.org/resource/doi:10.5061/dryad.77bv0).
