# Supplementary material for: Siderophore cooperation of the bacterium Pseudomonas fluorescens in soil
Source: Biol Lett. 2015 Feb;11(2):20140934. doi: 10.1098/rsbl.2014.0934 (PMC4360104; doi:10.1098/rsbl.2014.0934)
Supplement: Supplementary Information [file rsbl20140934supp1.docx]

SUPLEMENTARY MATERIAL

*1.Statistical analyses*

General linear mixed models (GLMs) with REML were employed to determine: *i*) whether *P. fluorescens*’s growth rates were affected by pyoverdine production with bacteria (producers, non-producers), soil (neutral, acidic) and time as fixed factors and the variance among replicates fitted as a random effect nested within bacteria and soil (figure 1a) and *ii*) whether growth rates of producers and non-producers were influenced by the presence of the other with “social environment” (monocultures, mixed), soil and time as fixed factors and replicate fitted as a random effect nested within social environment and soil (figure 1b). These models were run using JMP^®^ Pro version 11.0.0 (SAS Institute, Cary, NC, USA).

Two-way ANOVA tests were employed to assess whether the environment (static, shaken) and the initial non-producers frequency (0.01, 0.5 and 0.99) have effect on non-producers relative fitness (Figure 2a) and on the population final cell density (Figure 2b).

Student *t* tests (1-way) (corrected with the Benjamini-Hochberg method [1] for multiple comparisons) were performed to determine when the non-producers relative fitness was different from 1.

All data was checked for normality assumptions and log-transformed if necessary.

1. Benjamini, Y. & Hochberg,Y. (1995) Controlling the False Discovery Rate: A Practical and Powerful Approach to Multiple Testing. *Journal of the Royal Statistical Society*. 57, 289-300.
2. *Supplementary Figures*

**Figure S1**: Growth curves of *P. fluorescens* producers and non-producers in mono and mixed soil microcosms. (a) Producers and non-producers were grown as monocultures in acidic (solid line) and neutral (dashed line) soil microcosms and viable bacteria was monitored over a 30-day period. (b) Mixed cultures with a 50:50 equal ratio of each strain were inoculated into acidic (solid line) and neutral (dashed line) soil microcosms and cell numbers for each strain were assessed over a 30-day period. In both panels, producers and non-producers data point are represented as closed and open circles, respectively. Data points represent the average ± SEM of six independent replicates.

**Figure S2:** Relative fitness of non-producers in acidic and neutral soils. Bars represent the non-producers’ relative fitness (*v)* (mean ± SEM) at equal ratios of producers:non-producers after 30 days of growth in soil.
